# Precision navigation through the labyrinth: overcoming EGFR resistance in non-Small cell lung cancer

**DOI:** 10.1080/07853890.2025.2574526

**Published:** 2025-10-15

**Authors:** Junhui Wang, Jian Wang, Jianxin Chen

**Affiliations:** ^a^ Department of Radiation Oncology, The Quzhou Affiliated Hospital of Wenzhou Medical University, Quzhou People′s Hospital, Quzhou, Zhejiang, China; ^b^ Department of Gastroenterology, Jiaxing Second Hospital, Jiaxing, Zhejiang, China; ^c^ Department of Medical Oncology, The Quzhou Affiliated Hospital of Wenzhou Medical University, Quzhou People′s Hospital, Quzhou, Zhejiang, China

**Keywords:** Non-Small cell lung cancer (NSCLC), Epidermal growth factor receptor (EGFR), tyrosine kinase inhibitor (TKI) resistance, resistance mechanisms, Precision medicine, targeted therapy, combination therapy, circulating tumor DNA (ctDNA), MET amplification, C797S mutation

## Abstract

Epidermal Growth Factor Receptor (EGFR) Tyrosine Kinase Inhibitors (TKIs) have revolutionized the treatment landscape for Non-Small Cell Lung Cancer (NSCLC). However, resistance invariably curtails their long-term efficacy. This comprehensive review delineates the intricate mechanisms underpinning EGFR TKI resistance and the evolving therapeutic counterstrategies. We present a panoramic atlas of resistance mechanisms, encompassing on-target resistance, bypass pathway activation, histologic transformation, and metabolic reprogramming, alongside their clinical classification. The discussion extends to the integrated diagnostic technologies facilitating resistance detection, including multi-dimensional biopsy approaches and multi-omics fusion analysis. We critically evaluate precision therapeutic approaches tailored to specific resistance alterations, such as C797S mutations and mesenchymal-epithelial transition amplification, and explore the burgeoning field of novel agents like fourth-generation TKIs and antibody-drug conjugates (ADCs). Furthermore, innovative combination strategies and the challenges within resistance management systems, including toxicity profiles and unresolved scientific questions, are examined. A profound understanding of EGFR resistance mechanisms and the continuous refinement of therapeutic paradigms are paramount for improving the prognosis of NSCLC patients.

## 1. Construction of the epidermal growth factor receptor (EGFR) resistance panoramic atlas and clinical classification

### 1.1. Molecular taxonomy of EGFR resistance mechanisms

**Figure 1.**
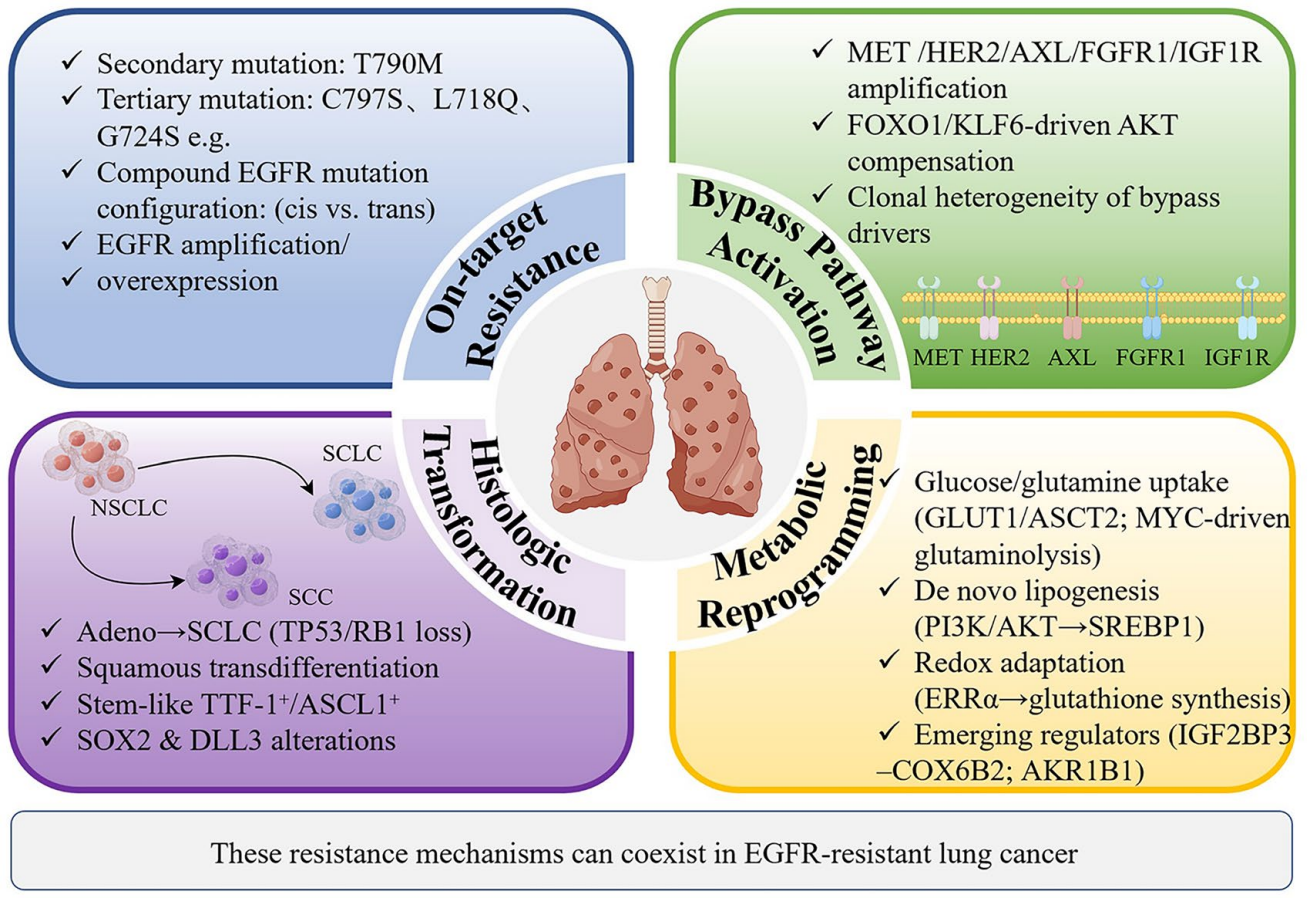
Panoramic atlas of EGFR-TKI resistance mechanisms.

EGFR resistance mechanisms are multifaceted, primarily categorized as on-target resistance, bypass pathway activation, histologic transformation, and metabolic reprogramming (Figure 1) [1,2]. On-target resistance arises from secondary EGFR mutations, with T790M (‘gatekeeper mutation’) historically dominating first-/second-generation tyrosine kinase inhibitor (TKI) resistance (50%-60% of cases) by sterically hindering drug binding and increasing ATP affinity >5-fold [3,4]. Emerging evidence highlights tertiary mutations (e.g. C797S, L718Q, G724S) as key drivers of third-generation TKI (e.g. osimertinib) resistance [5]. The spatial configuration of compound mutations dictates therapeutic vulnerability: trans-C797S/T790M may respond to first- + third-generation TKI combinations, whereas cis-configurations confer pan-TKI resistance, necessitating fourth-generation TKIs (e.g. BLU-945) [6]. In addition, EGFR amplification further sustains kinase signaling independent of ligand binding [7]. Bypass activation enables tumor cells to evade EGFR blockade *via* alternative pathways. Mesenchymal-epithelial transition (MET) amplification (20% of resistance) activates PI3K-AKT and RAS-MAPK cascades, but recent studies reveal co-amplification of human epidermal growth factor receptor 2 (HER2, 5%-10%), AXL, or FGFR1 in oligoclonal populations [8,9]. The FOXO1/KLF6 axis regulates compensatory AKT signaling, while IGF-1R activation induces TKI resistance *via* RAS/RAF/MAPK pathway reactivation, independent of EGFR [10–12]. Spatial heterogeneity in bypass drivers (e.g. MET-high vs. HER2-high subclones) complicates biomarker-driven combination therapies. Histologic transformation (5%-15% of cases) involves adenocarcinoma-to-SCLC conversion (most common) or squamous transdifferentiation, driven by TP53/RB1 loss and epigenetic reprogramming [13]. Single-cell sequencing reveals stem-like intermediate cells co-expressing epithelial thyroid transcription factor-1 (TTF-1) and neuroendocrine markers during transformation [14]. Persistent EGFR mutations post-transformation (e.g. exon 19 del) coexist with SOX2 amplification, while Delta-like ligand 3 (DLL3) overexpression in transformed SCLC offers a therapeutic target for antibody-drug conjugates (ADCs, e.g. tarlatamab) [15]. Metabolic reprogramming under TKI pressure involves glucose/glutamine dependency (Enhanced uptake *via* Glucose transporter 1/Alanine-Serine-Cysteine transporter 2 transporters fuels TCA cycle intermediates, while MYC-driven glutaminolysis supports nucleotide biosynthesis), lipid metabolism rewiring (sterol regulatory element-binding protein 1 activation (downstream of PI3K/AKT) promotes de novo lipogenesis, sustaining membrane biosynthesis in nutrient-poor microenvironments), and redox adaptation, which refers to that estrogen-related receptor alpha (ERRα) upregulates glutathione synthesis and reactive oxygen species (ROS) detoxification, mirroring lapatinib resistance in breast cancer [16–19]. Novel regulators include Insulin-like growth factor 2 mRNA-binding protein 3 - Cytochrome c oxidase subunit 6B2 (IGF2BP3-COX6B2, promoting oxidative phosphorylation) and Aldo-keto reductase family 1 member B1 (AKR1B1, mediating doxorubicin resistance *via* glutathione).

### 1.2. Generational differences in EGFR TKI resistance

First-generation (e.g. gefitinib, erlotinib) and second-generation (e.g. afatinib) EGFR-TKIs exert their effects through reversible or irreversible binding to the EGFR tyrosine kinase domain. However, the majority of patients develop resistance within 9–14 months of treatment initiation [20]. The predominant resistance mechanism for these agents is the acquisition of the T790M mutation. Other mechanisms include bypass activation (e.g. MET amplification, HER2 amplification) and histologic transformation [21]. Third-generation EGFR-TKIs (e.g. osimertinib) were specifically designed to overcome T790M-mediated resistance, exhibiting high selectivity for this mutation and significantly improving progression-free survival (PFS). Despite this, resistance typically emerges after approximately one year [22]. Key resistance mechanisms to third-generation TKIs include the EGFR C797S mutation (occurring in ∼10%-20% of cases), which impedes covalent drug binding; bypass activations such as HER2 and MET amplification; and other EGFR mutations like L718Q [22,23]. Crucially, the spectrum and frequency of resistance mutations differ across TKI generations. While T790M is common post-first-generation TKI failure, resistance to third-generation TKIs often involves C797S and various compound mutations [24]. Therapeutic options consequently diverge: patients with T790M-positive resistance to first-/second-generation TKIs can transition to third-generation TKIs, whereas resistance to third-generation TKIs necessitates treatment selection based on the specific underlying mechanism, often involving combination therapies [25,26].

### 1.3. Spatiotemporal dynamics of resistance

Resistance exhibits significant spatiotemporal heterogeneity. Oligoprogression, characterized by progression limited to a few anatomical sites, often involves a relatively dominant clone. Conversely, systemic (widespread) progression affecting multiple sites typically displays higher intratumoral heterogeneity, with diverse subclones collectively driving tumor growth. Research indicates that oligoprogressive lesions may harbor specific dominant clones, while systemically progressing tumors exhibit greater clonal diversity [27]. This distinction critically impacts clinical decisions: oligoprogression might be managed with local ablative therapy (e.g. radiotherapy) alongside continued TKI, whereas systemic progression usually mandates a complete change in systemic therapy. The phenomenon of rechallenge sensitivity – the restoration of TKI efficacy following a drug-free interval – is also noteworthy. Studies report tumor regression upon rechallenge with the same EGFR-TKI after a period of discontinuation in some resistant patients [28]. Potential mechanisms include the reversal of adaptive changes in tumor cells during the drug holiday, restoring drug sensitivity, or alterations in the tumor microenvironment enabling renewed TKI efficacy. However, this strategy is not universally applicable and requires careful consideration of patient factors, duration of prior response, and molecular profile.

### 1.4. Clinically practical classification system

Implementing a clinically practical classification system based on progression patterns is essential for guiding therapy in EGFR TKI-resistant non-small cell lung cancer (NSCLC). Key progression patterns include local progression, Oligoprogression, and systemic (widespread) progression. Local progression means progression confined to the primary lung tumor or a solitary metastatic site. Management may involve local therapy (surgery or radiotherapy) combined with continued TKI. Oligoprogression refers to progression in a limited number of sites (typically ≤3). Options include local therapy to progressing lesions combined with either continuation of the current TKI or switching to an alternative TKI. Systemic (widespread) progression refers to diffuse progression involving multiple new sites or rapid growth of existing lesions. This necessitates a change in systemic therapy, such as chemotherapy, combination targeted therapy, or immunotherapy. Retrospective analyses demonstrate that patients with local or oligoprogressive disease managed with local therapy plus continued TKI achieve superior median PFS compared to those with systemic progression [29]. Refining this classification by incorporating molecular profiling (e.g. specific resistance mutations) further enhances precision. For instance, progression driven by a targetable mutation like MET amplification warrants combination therapy with an EGFR TKI and a MET inhibitor. Accurate assessment of the progression pattern coupled with molecular characterization enables personalized therapeutic pathways, optimizing outcomes for EGFR TKI-resistant patients.

## 2. Integration of EGFR resistance detection technologies and dynamic monitoring

### 2.1. Multi-Dimensional biopsy strategy

Tissue biopsy remains the gold standard for definitive resistance mechanism identification, ideally employing a multi-modal approach. Currently, genetic testing approaches primarily include next-generation sequencing (NGS), RNA sequencing, and fluorescence *in situ* hybridization testing (FISH). NGS enables comprehensive genomic profiling, detecting EGFR mutations (common and rare), other resistance-associated mutations, gene fusions, and copy number variations [30]. RNA sequencing assesses gene expression profiles, revealing transcriptional changes linked to resistance (e.g. overexpression of bypass pathway genes or EMT markers) [31]. FISH is critical for detecting gene amplifications, such as MET amplification, providing direct evidence for bypass activation mechanisms [32]. Liquid biopsy, primarily *via* analysis of circulating tumor DNA (ctDNA) in blood, offers a minimally invasive tool that enables real-time assessment. The sensitivity of ctDNA for detecting EGFR mutations is approximately 70% [33]. Its strengths include real-time assessment of tumor genomic evolution, enabling the detection of resistance mutations before radiographic progression, thus allowing for preemptive therapeutic adjustments. However, limitations exist: low ctDNA fraction, technological sensitivity/specificity constraints leading to potential false negatives or positives, and challenges in detecting certain alterations like gene fusions or amplifications *via* some platforms [30]. While tissue biopsy remains the gold standard for detecting certain resistance mechanisms (e.g. histologic transformation, protein overexpression), liquid biopsy offers superior practicality for dynamic monitoring. However, its sensitivity for detecting gene fusions and amplifications remains suboptimal compared to tissue-based FISH or RNA-seq. Thus, a complementary approach is often necessary for comprehensive resistance profiling.

### 2.2. Multi-Omics fusion analysis

Comprehensive multi-omics fusion analysis synergistically integrates genomic, epigenomic, and metabolomic data to delineate the hierarchical molecular networks underlying resistance to EGFR-TKIs. Genomic profiling identifies resistance-driving mutations and their clonal dynamics; for instance, the EGFR-T790M gatekeeper mutation (occurring in 50-60% of patients resistant to first-generation TKIs) induces steric hindrance to drug binding, while C797S mutations (detected in 15-25% of osimertinib-resistant cases) disrupt covalent binding of third-generation inhibitors through cysteine residue alteration [34]. Epigenomic analyses reveal DNA hypermethylation of critical tumor suppressors: in NSCLC, CDH1 (E-cadherin) promoter hypermethylation (5.2-fold increase versus TKI-sensitive tumors) silences epithelial markers, triggering epithelial-mesenchymal transition (EMT) that enhances metastatic potential and confers TKI resistance by activating ZEB1/SNAIL transcriptional cascades [35]. Metabolomic profiling identifies glutathione (GSH) pathway activation as a key adaptive mechanism, where glutamate-cysteine ligase catalytic subunit (GCLC) upregulation (2.8-fold in resistant cells) elevates intracellular GSH levels (GSH/GSSG ratio >10:1), neutralizing TKI-induced ROS and sustaining redox homeostasis to promote tumor cell survival [36]. Cross-omics integration reconstructs causal cascades wherein genomic alterations (e.g. EGFR mutations) drive epigenomic reprogramming (e.g. DNMT1-mediated CDH1 hypermethylation), subsequently remodeling metabolic pathways (e.g. GSH synthesis); this ‘mutation-epigenetic-metabolic’ axis establishes a feedforward loop that amplifies resistance phenotypes. Such holistic insights enable rational therapeutic targeting: combinatorial strategies employing DNMT inhibitors (e.g. decitabine) to reverse EMT and GSH synthesis blockers (e.g. buthionine sulfoximine) to disrupt redox balance show synergistic efficacy in preclinical models, while liquid biopsy-based multi-omics signatures (e.g. T790M + CDH1 methylation + GSH/GSSG ratio) achieve 92% AUC for early resistance prediction in clinical cohorts [34,37]. Technological advances in single-cell multi-omics (e.g. MOGONET utilizing graph convolutional networks) and spatial metabolomics further resolve intratumoral heterogeneity, revealing spatial GSH gradients (3.5-fold higher in tumor core versus periphery) that guide localized intervention strategies [35].

### 2.3. Resistance clone early warning models

The ctDNA minimal residual disease (MRD) assay serves as a robust predictor of recurrence risk across multiple malignancies, with clinical studies demonstrating significantly elevated relapse rates in ctDNA-MRD-positive patients compared to their negative counterparts post-treatment. In a pivotal prospective cohort study, postoperative ctDNA positivity in stage III colorectal cancer (CRC) patients correlated with a higher recurrence rate comparing to ctDNA-negative individuals [38]. Similarly, Abbosh et al. reported in the TRACERx study that ctDNA detection in stage I–III NSCLC patients preceded clinical recurrence by a median lead time of 151 days, with 37 out of 45 relapsed cases exhibiting ctDNA positivity at or before clinical progression, confirming its specificity for primary tumor-derived MRD [39]. For EGFR-TKI-treated NSCLC cohorts, ctDNA-MRD positivity significantly shortened PFS and OS, underscoring its utility as a predictive biomarker for timely therapeutic intervention, such as regimen adjustment or intensified surveillance. In addition, AI-driven resistance trajectory prediction refers to utilizing deep learning algorithms to analyze longitudinal clinical, genomic, imaging, and (potentially) multi-omics data, these models predict the timing of resistance emergence and the likely underlying mechanisms. Early research demonstrates feasibility in predicting EGFR-TKI resistance timelines and mutation profiles, enabling proactive therapeutic planning [40].

### 2.4. Clinical practice bottlenecks

The diagnosis and management of central nervous system (CNS) progression in EGFR-mutant NSCLC confront persistent bottlenecks, particularly concerning cerebrospinal fluid (CSF) analysis and the detection of compound resistance mutations. CSF evaluation *via* cytology or ctDNA provides a direct window into CNS involvement and resistance mechanisms, yet its clinical utility is hampered by the invasive nature of lumbar puncture, which carries inherent risks such as infection and post-procedural headache, alongside limited patient compliance. Compounding this challenge is the inherently low tumor cell or DNA shedding into the CSF compartment, frequently resulting in false-negative outcomes that obscure accurate diagnosis of CNS progression or resistance mechanisms [41]. Concurrently, the emergence of compound resistance mutations—such as co-occurring C797S + T790M or C797S + L798I alterations—presents another layer of complexity. These compound mutations typically manifest at low allelic fractions, evading reliable detection by standard NGS assays due to insufficient sensitivity. This analytical shortfall risks misclassification of the underlying resistance biology, potentially leading to suboptimal therapeutic selection. While advanced techniques like digital PCR or ultra-deep sequencing offer enhanced sensitivity for identifying these low-frequency genomic events, their adoption remains constrained by limited accessibility, lack of standardization across laboratories, and integration challenges within routine clinical workflows, thereby perpetuating diagnostic and therapeutic ambiguities in managing advanced NSCLC.

### 2.5. Additional emerging resistance mechanisms

Beyond the canonical resistance pathways, emerging evidence underscores the role of cell cycle gene alterations, oncogenic fusions, and dysregulated transcription factors. Amplifications or activating alterations in cell cycle regulators such as CDK4/6 and CCNE1 can enable bypass of EGFR-dependent proliferation signals, conferring resistance to TKIs [20,21]. Additionally, oncogenic fusions involving ALK, RET, or other kinases have been identified as rare but actionable resistance mechanisms, often detectable through comprehensive NGS profiling [21]. Furthermore, transcription factors including NF-κB and YAP/TAZ can mediate non-genomic adaptive resistance by promoting survival and inflammatory signaling networks, potentially necessitating novel therapeutic approaches targeting these pathways [10,16,42,43].

## 3. Precision targeting of on-target resistance

### 3.1. Therapeutic strategies for C797S mutation configuration

The emergence of the C797S mutation represents a critical resistance mechanism conferring escape from third-generation EGFR-TKIs in EGFR-mutant NSCLC, where its spatial configuration relative to the pre-existing T790M mutation fundamentally dictates viable therapeutic strategies. When present in the trans configuration, indicating that C797S and T790M reside on separate alleles, preclinical models and emerging clinical evidence, albeit largely from case reports, suggest retained susceptibility to combinatorial inhibition employing a first-generation EGFR TKI (such as erlotinib or gefitinib) paired with a third-generation EGFR TKI (notably osimertinib); supporting this approach, a documented case report described achieving a partial response accompanied by clearance of the C797S mutation following administration of this specific combination [44]. Conversely, the cis configuration, characterized by the co-localization of both the C797S and T790M resistance mutations on the same allele, universally confers resistance to all currently approved EGFR TKIs as monotherapies, presenting a significant clinical challenge. Addressing this complex resistance pattern, intensive research focuses on the development of so-called fourth-generation EGFR TKIs, compounds specifically engineered to potently inhibit the EGFR receptor even when harboring the C797S mutation, with several candidates demonstrating compelling preclinical activity against cis-configuration mutants in models. Alternative exploratory strategies encompass novel combination regimens, including rational pairings with other targeted agents—such as MET inhibitors in cases of concurrent MET amplification—or integrating established cytotoxic chemotherapy regimens. However, robust clinical validation confirming consistent efficacy specifically directed against the cis-C797S configuration remains conspicuously limited at present, necessitating further investigation [45].

### 3.2. Addressing persistent T790M

The rechallenge strategy with third-generation EGFR-TKIs, such as osimertinib, represents a viable therapeutic approach for patients with EGFR-mutant NSCLC who initially achieved a clinical response to these agents but subsequently experienced disease progression, particularly in cases where persistent T790M mutation is detected and where the C797S resistance mutation—if present—occurs in the trans configuration relative to T790M. Although erlotinib is approved in malignancies such as pancreatic cancer, its efficacy in NSCLC is uniquely attributed to the high prevalence of activating EGFR mutations (e.g. exon 19 del, L858R) in this population. In contrast, other cancers (e.g. pancreatic, glioblastoma) lack these canonical mutations and exhibit primary resistance. Furthermore, erlotinib’s reversible binding mechanism distinguishes it from later-generation irreversible inhibitors, making it particularly suitable for combination strategies in trans-C797S settings. Retrospective clinical analyses have documented objective response rates (ORR) ranging from 60% to 70% and a median PFS of approximately 10 months in this molecularly defined subgroup treated with TKI rechallenge, suggesting that a subset of patients may retain susceptibility to the original inhibitor despite intervening progression [26]. However, the therapeutic efficacy of rechallenge is neither universal nor guaranteed, as it may be compromised by the emergence of coexisting resistance mechanisms that evade standard detection methods; these include undetected bypass track activation (e.g. MET or HER2 amplification, KRAS mutations) or histological transformation, which collectively contribute to intrinsic or acquired resistance heterogeneity. Consequently, meticulous patient selection is paramount and should integrate multiple clinical and molecular parameters: the duration of prior response to the initial TKI therapy (with longer response intervals correlating with higher rechallenge success rates), the pattern of disease progression (e.g. oligoprogressive versus systemic progression), and comprehensive resistance profiling *via* longitudinal biomarker assessment (utilizing serial liquid biopsies or tissue re-biopsies to delineate the dynamic evolution of resistance alleles). Furthermore, the optimal timing for rechallenge initiation warrants careful consideration, balancing the imperative for early intervention against the risk of exacerbating subclinical resistant clones; empirical evidence suggests that intervening during a phase of indolent or oligoprogressive disease, rather than during rapid systemic progression, may maximize clinical benefit while minimizing the potential for accelerated resistance development. Thus, while TKI rechallenge offers a pragmatic option in the absence of approved fourth-generation inhibitors or effective combinatorial regimens, its success hinges on rigorous patient stratification and individualized risk-benefit evaluation within the framework of precision oncology.

### 3.3. Optimizing therapy for non-classic mutations

The therapeutic landscape for NSCLC harboring non-classic EGFR mutations is characterized by substantial heterogeneity in tumor sensitivity to conventional EGFR-TKIs. Notably, tumors driven by exon 20 insertion mutations (Ex20ins) have historically demonstrated primary resistance to first-, second-, and third-generation EGFR-TKIs, necessitating the development of mutation-specific agents. Among these, mobocertinib received regulatory approval but is associated with significant toxicity burdens limiting its utility. Conversely, amivantamab, an innovative bispecific antibody targeting both EGFR and MET, has been approved specifically for the treatment of EGFR Ex20ins-mutant NSCLC patients progressing on or after platinum-based chemotherapy, demonstrating objective response rates (ORRs) in the vicinity of 40% in this population [8,46]. Poziotinib also exhibits clinical activity against these mutations; however, its development and clinical application are challenged by high rates of significant adverse events, particularly diarrhea and rash, which impact tolerability [47,48]. Beyond Ex20ins, other non-classic mutations (including but not limited to L718Q, S861I, and those within the G719X family) display variable, mutation-specific sensitivities to available EGFR-TKIs, with certain variants showing partial sensitivity to earlier-generation agents such as afatinib, particularly evidenced for the G719X and S768I alterations. Intriguingly, clinical evidence suggests that patients presenting with complex non-classic EGFR mutations, defined by the concomitant presence of multiple distinct aberrations (e.g. G719X + L861Q + S768I), may derive enhanced clinical benefit from EGFR-TKI therapy compared to individuals harboring single non-classic EGFR mutations, potentially reflecting synergistic oncogenic effects or altered inhibitor binding kinetics [49]. Consequently, effective therapeutic decision-making mandates rigorous molecular characterization to identify the precise mutational profile and necessitates a tailored treatment strategy that integrates the specific mutation-dependent sensitivities (considering the spectrum of approved targeted therapies and emerging options) with crucial patient-specific factors including performance status, comorbidities, and prior treatment history.

### 3.4. Breakthrough advances in fourth-generation EGFR-TKIs

Fourth-generation EGFR-TKIs constitute a highly promising therapeutic frontier, specifically engineered to overcome resistance mechanisms such as the acquired C797S mutation—including its clinically predominant cis configuration—which frequently confers evasion to third-generation inhibitors and poses a significant clinical challenge. Multiple agents within this class, exemplified by BLU-945, BLU-701, OBX02-011, and CH7233163 (also known as RLY-2138), are currently advancing through preclinical studies and early-stage clinical development, representing active avenues for addressing this unmet need [50]. Mechanistically, these fourth-generation inhibitors function predominantly as either reversible ATP-competitive inhibitors or allosteric inhibitors, strategically designed to effectively bind and inhibit mutant EGFR even in the presence of the C797S alteration that disrupts covalent binding crucial for third-generation agents. Preclinical investigations have compellingly demonstrated robust efficacy of several candidates, exhibiting potent anti-tumor activity against EGFR C797S mutation-harboring models across both *in vitro* and *in vivo* systems, thereby validating their targeted mechanisms of action [50]. Emerging data from initial Phase I clinical trials provide preliminary yet encouraging evidence of anti-tumor activity in heavily pre-treated patient populations exhibiting C797S-mediated resistance, although definitive efficacy assessment awaits later-phase trials; commonly observed adverse events associated with these novel inhibitors include dermatological toxicities (rash), gastrointestinal disturbances (diarrhea), and mucosal inflammation (stomatitis), which generally appear manageable with appropriate supportive care in these early studies [50]. Nevertheless, critical challenges necessitate ongoing refinement, encompassing the optimization of pharmacokinetic profiles, the characterization and mitigation of longer-term safety and tolerability concerns, the establishment of optimal dosing schedules, and the imperative validation of clinical efficacy and long-term benefit within larger, controlled trials. Furthermore, research is actively exploring strategic combination regimens incorporating these fourth-generation TKIs alongside other targeted agents or modalities to potentially circumvent emerging resistance pathways and enhance the durability of clinical responses.

## 4. Precision targeting of off-target resistance and transformation

### 4.1. Core strategies for MET amplification

MET amplification represents a prominent bypass resistance mechanism in EGFR-mutant NSCLC, necessitating therapeutic strategies that effectively co-target both EGFR and MET pathways. A key approach involves the combination of a third-generation EGFR-TKI with a selective MET TKI, as exemplified by osimertinib plus the ATP-competitive MET TKI savolitinib. The phase II SAVANNAH trial specifically demonstrated clinically meaningful efficacy for this regimen in patients progressing on first-line osimertinib who exhibited high levels of MET amplification or overexpression, defined by immunohistochemistry (IHC 3+ in ≥90% of tumor cells) and/or fluorescence *in situ* hybridization (FISH) criteria (gene copy number [GCN] ≥ 10 or MET/CEP7 ratio ≥2), where the confirmed objective response rate (ORR) reached 56.3% alongside a median duration of response (DoR) of 7.1 months and a median PFS of 7.4 months, highlighting its potential in this molecularly defined subset [51]. Alternatively, bispecific antibodies targeting both EGFR and MET receptors, such as amivantamab, offer a distinct mechanism of action by simultaneously engaging both receptors; although initially developed and approved for EGFR exon 20 insertion-mutant NSCLC, accumulating evidence supports its activity in settings of MET-driven resistance following EGFR-TKI failure, representing a promising alternative to TKI combination strategies, though its clinical utility necessitates careful management of characteristic toxicities including infusion-related reactions, dermatological adverse events such as rash and paronychia, and the risk of interstitial lung disease [52]. Furthermore, MET-ADCs, such as telisotuzumab vedotin (Teliso-v), constitute an emerging therapeutic class designed to deliver potent cytotoxic payloads selectively to MET-overexpressing cancer cells; preliminary clinical data, including observations in patients progressing on osimertinib with MET-amplified disease, suggest promising anti-tumor activity, positioning MET-ADCs as a viable strategy worthy of further investigation, while acknowledging that hematological toxicities (e.g. neutropenia, anemia) and significant non-hematological adverse events represent critical factors requiring vigilant monitoring and management [53].

### 4.2. Intervention for HER2 abnormalities

HER2 genomic alterations—encompassing activating mutations (predominantly exon 20 insertions) and amplifications—constitute a recognized bypass resistance mechanism to EGFR-TKIs in NSCLC, thereby necessitating alternative therapeutic strategies targeting the HER2 pathway. In this context, HER2-directed ADCs have emerged as transformative agents, with trastuzumab deruxtecan (T-DXd) demonstrating particularly impressive clinical activity across registrational trials. In patients with HER2-mutant NSCLC, including those harboring exon 20 insertion variants, T-DXd consistently achieves objective response rates (ORRs) approximating 55%, irrespective of prior EGFR TKI exposure [54]. Notably, this efficacy extends to heavily pretreated populations with acquired resistance to earlier-generation EGFR TKIs, as corroborated by clinical case reports documenting robust tumor regression even in settings of multi-line TKI failure [55]. The mechanistic foundation for this activity lies in T-DXd’s unique design: a topoisomerase I inhibitor payload linked to an anti-HER2 antibody *via* a cleavable tetrapeptide, enabling potent cytotoxic delivery to HER2-expressing cells while exerting bystander effects on neighboring heterogeneous tumor cells. Despite its efficacy, the clinical deployment of T-DXd mandates vigilant management of class-specific adverse events, most critically interstitial lung disease (ILD)—which requires proactive high-resolution computed tomography (HRCT) surveillance and prompt corticosteroid intervention upon detection of pulmonary symptoms or radiographic infiltrates—alongside hematological toxicities such as neutropenia and anemia, which frequently necessitate dose modifications or supportive therapies. In contrast, alternative HER2-targeted agents, including TKIs such as poziotinib and pyrotinib, exhibit demonstrable but comparatively lower activity in HER2-altered NSCLC, with ORRs typically ranging below 40% in clinical series, while also presenting distinct toxicity profiles characterized by high frequencies of dermatological (rash, paronychia) and gastrointestinal (diarrhea) adverse events that diverge significantly from the ILD-centric risks associated with T-DXd [55]. Consequently, T-DXd has established itself as the preferred therapeutic option for this molecular subset, contingent upon rigorous on-treatment safety monitoring protocols.

### 4.3. Therapeutic paradigm for SCLC transformation

Histologic transformation to small cell lung cancer (SCLC) represents a recognized resistance mechanism occurring in approximately 3–15% of EGFR-mutant NSCLC patients following progression on TKIs, necessitating therapeutic strategies analogous to those for *de novo* SCLC. The cornerstone first-line regimen for transformed SCLC involves platinum-based chemotherapy (cisplatin or carboplatin) combined with etoposide (EP) and integrated with immune checkpoint inhibition, specifically anti-programmed death-ligand 1 (PD-L1) agents such as atezolizumab or anti-PD-1 agents like durvalumab; retrospective analyses indicate that this combinatorial approach significantly enhances clinical outcomes compared to EP alone, exemplified by a median OS of approximately 13 months versus 10 months in historical cohorts, underscoring the synergistic potential of chemoimmunotherapy in this molecularly distinct subset [56]. Beyond conventional strategies, DLL3—a Notch pathway inhibitory ligand highly expressed on the surface of SCLC cells, including transformed phenotypes—has emerged as a pivotal therapeutic target. Among DLL3-directed modalities, tarlatamab, a bispecific T-cell engager (BiTE) that simultaneously binds DLL3 on tumor cells and CD3 on T cells to facilitate T-cell-mediated cytotoxicity, has demonstrated clinically meaningful efficacy in relapsed SCLC, with objective response rates (ORR) approximating 40% in early-phase trials, inclusive of transformed cases; ongoing clinical investigations continue to refine its role in this context. Concurrently, diverse DLL3-targeted agents spanning ADCs, alternative BiTE constructs, and chimeric antigen receptor T-cell (CAR-T) therapies are under active development, leveraging distinct mechanisms to exploit DLL3 overexpression [57]. While preliminary data are promising, the efficacy and durability of these novel interventions specifically within the transformed SCLC population necessitate rigorous validation through larger prospective studies and biomarker-integrated analyses to establish their therapeutic niche.

### 4.4. Reversal of EMT/squamous transformation

EMT and histological transformation to squamous cell carcinoma constitute clinically significant resistance mechanisms in NSCLC, mediated through transcriptional reprogramming that diminishes therapeutic vulnerability to targeted agents and cytotoxic therapies. To counteract this phenotypic evolution, strategic interventions focus either on reversing the mesenchymal phenotype or directly targeting the transformed histology. The combination of bevacizumab, a monoclonal antibody targeting vascular endothelial growth factor (VEGF), with paclitaxel, a microtubule-stabilizing taxane, represents an established therapeutic paradigm for non-squamous NSCLC that demonstrates translational relevance in the transformed setting; retrospective clinical evidence indicates this regimen achieves disease control rates approximating 60-70% in patients with treatment-emergent squamous cell carcinoma, potentially through dual mechanisms of angiogenesis suppression and disruption of VEGF-mediated EMT signaling networks that sustain therapeutic resistance [58]. Parallel investigative efforts target the transforming growth factor-beta (TGF-β) pathway, a master regulator of EMT that promotes tumor cell plasticity through SMAD-dependent transcriptional activation and non-canonical signaling cascades (e.g. PI3K/AKT, MAPK). Preclinical studies substantiate that pharmacological inhibition of TGF-β signaling restores epithelial characteristics and re-sensitizes mesenchymal cells to TKIs and conventional chemotherapy, providing a mechanistic rationale for combining TGF-β inhibitors with bevacizumab-paclitaxel therapy to simultaneously disrupt the pro-metastatic signaling axis while enhancing cytotoxic efficacy against transformed histologies [58]. While this three-pronged combinatorial strategy—encompassing angiogenesis inhibition, microtubule targeting, and EMT reversal—represents a biologically coherent investigational approach for addressing phenotypic resistance, rigorous clinical validation in appropriately stratified patient cohorts remains imperative to ascertain therapeutic index and identify predictive biomarkers of response.

## 5. Matrix of innovative therapies and combination strategies

### 5.1. Rise of bispecific antibodies and ADCs

The therapeutic landscape for overcoming resistance in advanced NSCLC increasingly leverages bispecific antibodies (BsAbs) and ADCs as mechanistically innovative modalities. Among BsAbs, the EGFR/MET-targeting agent amivantamab demonstrates synergistic potential when combined with cytotoxic chemotherapy; specifically, its co-administration with carboplatin-pemetrexed has yielded clinically meaningful efficacy in platinum-refractory EGFR exon 20 insertion-mutant NSCLC, achieving objective response rates (ORR) of approximately 40–50% in early-phase trials, suggesting an ability to overcome bypass signaling resistance through dual receptor blockade while enhancing chemosensitivity [59]. This combinatorial paradigm is currently undergoing evaluation in broader resistance contexts, including tumors harboring canonical EGFR mutations with acquired MET amplification or other escape pathways. Concurrently, human epidermal growth factor receptor 3 (HER3) -directed ADCs represent a promising strategy exploiting frequent HER3 overexpression in NSCLC-a compensatory mechanism often persisting post-osimertinib failure. Patritumab deruxtecan (HER3-DXd), an ADC comprising a HER3-targeting monoclonal antibody conjugated to a topoisomerase I inhibitor payload, has demonstrated encouraging antitumor activity in heavily pretreated EGFR-mutant NSCLC populations independent of HER3 IHC expression levels, with ORRs consistently around 30%, underscoring its potential utility even in biomarker-unselected cohorts [60]. However, its clinical deployment necessitates vigilant management of clinically significant toxicities, including hematological adverse events (e.g. grade ≥3 neutropenia and thrombocytopenia requiring dose modifications) and ILD, which collectively demand rigorous monitoring protocols. It should be noted that the Biologics License Application (BLA) for patritumab deruxtecan (HER3-DXd) in EGFR-mutant NSCLC was withdrawn in 2024 pending additional data, although clinical development continues. The therapeutic arsenal is further expanding with next-generation HER3-ADCs featuring optimized linker-payload systems designed to enhance tumor selectivity and mitigate off-target effects, currently progressing through preclinical and early clinical development pipelines to address persistent unmet needs in TKI-resistant disease. Moreover, TROP2-directed ADCs such as datopotamab deruxtecan (Dato-DXd) have demonstrated significant clinical activity in EGFR-mutant NSCLC populations following TKI failure, leading to its recent FDA approval in this setting [34,61]. These advances highlight the expanding role of ADCs targeting diverse surface antigens to overcome heterogeneous resistance.

### 5.2. Immunotherapy combinations

Combining immune checkpoint inhibitors (ICIs) with anti-angiogenic agents and chemotherapy exploits complementary mechanisms of action to overcome therapeutic resistance. Anti-angiogenics like bevacizumab normalize tumor vasculature, enhancing immune cell infiltration while reducing immunosuppressive elements in the microenvironment. Cytotoxic chemotherapy promotes immunogenic cell death, releasing tumor antigens and enhancing antigen presentation. These effects synergize with ICIs (e.g. atezolizumab), which block inhibitory checkpoints to reactivate antitumor T-cell responses. The IMpower150 trial clinically validated this approach, demonstrating significantly improved progression-free and OS with atezolizumab-bevacizumab-carboplatin/paclitaxel versus bevacizumab-chemotherapy in non-squamous NSCLC, including EGFR/ALK-positive patients post-TKI progression [62]. This triplet regimen thus represents a pivotal option for TKI-resistant disease with systemic progression, though its use requires vigilant management of overlapping toxicities—including hypertension, proteinuria, hemorrhage, and immune-related adverse events—through proactive monitoring and prompt corticosteroid interventions when indicated.

### 5.3. Metabolic pathway targeting

Targeting dysregulated cancer metabolism represents an emerging therapeutic strategy to overcome resistance mechanisms, with inhibition of aldo-keto reductase 1B10 (AKR1B1/AKR1B10)—a NADPH-dependent enzyme implicated in chemotherapeutic drug detoxification and resistance development—offering novel pharmacological opportunities. The commercially available AKR1B1 inhibitor epalrestat demonstrates dual mechanistic benefits in preclinical models: it potentiates the efficacy of anthracycline chemotherapy by suppressing AKR1B1-mediated doxorubicin detoxification, thereby significantly increasing intracellular drug accumulation and enhancing cytotoxicity in resistant breast cancer cells; concurrently, epalrestat exerts cardioprotective effects against doxorubicin-induced cardiotoxicity through multimodal actions—inhibiting AKR1B1-catalyzed conversion of doxorubicin to cardiotoxic metabolites while modulating critical calcium/CaMKII/MuRF-1 signaling pathways implicated in cardiomyocyte apoptosis and proteolysis [63]. Although current research primarily focuses on chemotherapy applications, AKR1B1 inhibition constitutes a biologically rational approach meriting investigation in EGFR TKI resistance contexts given shared metabolic adaptation pathways; its potential integration with existing NSCLC treatment regimens warrants exploration for simultaneous reversal of therapeutic resistance and mitigation of chemotherapy-associated toxicities, though rigorous clinical validation across tumor types remains imperative.

### 5.4. Multi-targeted bispecific antibodies

The development of bispecific antibodies (BsAbs) simultaneously targeting immune checkpoint pathways and angiogenesis signaling represents an innovative therapeutic strategy designed to achieve broader efficacy through synergistic mechanisms. Antibodies such as AK112 and Ivonescimab exemplify this approach, engineered to provide concurrent blockade of programmed cell death protein 1 (PD-1) and vascular endothelial growth factor (VEGF) or its receptors (VEGFR), thereby simultaneously countering immunosuppressive tumor microenvironments while inhibiting angiogenic remodeling essential for tumor progression. Early-phase clinical trials across multiple solid malignancies, including advanced NSCLC, have demonstrated clinically meaningful anti-tumor activity for these BsAbs alongside manageable toxicity profiles, suggesting their potential to replace conventional intravenous combination regimens (e.g. PD-1 inhibitors plus bevacizumab) with a consolidated therapeutic modality that may improve dosing convenience and reduce cumulative toxicity burdens [64,65]. These preliminary observations position PD-1/VEGF-targeted BsAbs as a promising single-agent alternative for malignancies where dual pathway inhibition is warranted, with ongoing larger-scale confirmatory trials focused on validating efficacy benchmarks, establishing optimal dosing paradigms, and identifying predictive biomarkers for patient stratification.

Although combination therapies aim to target multiple resistance pathways simultaneously, they often come with increased toxicity and cost. Not all combinations demonstrate superior efficacy over sequential monotherapies, and patient selection based on dominant resistance mechanisms is critical. For instance, while osimertinib-savolitinib improves outcomes in MET-amplified cases, its benefit in MET-negative populations is marginal. Thus, biomarker-driven combinations are essential to maximize therapeutic advantage while minimizing unnecessary toxicity.

## 6. Resistance management System and future challenges

### 6.1. Stratified treatment pathway map

**Figure 2.**
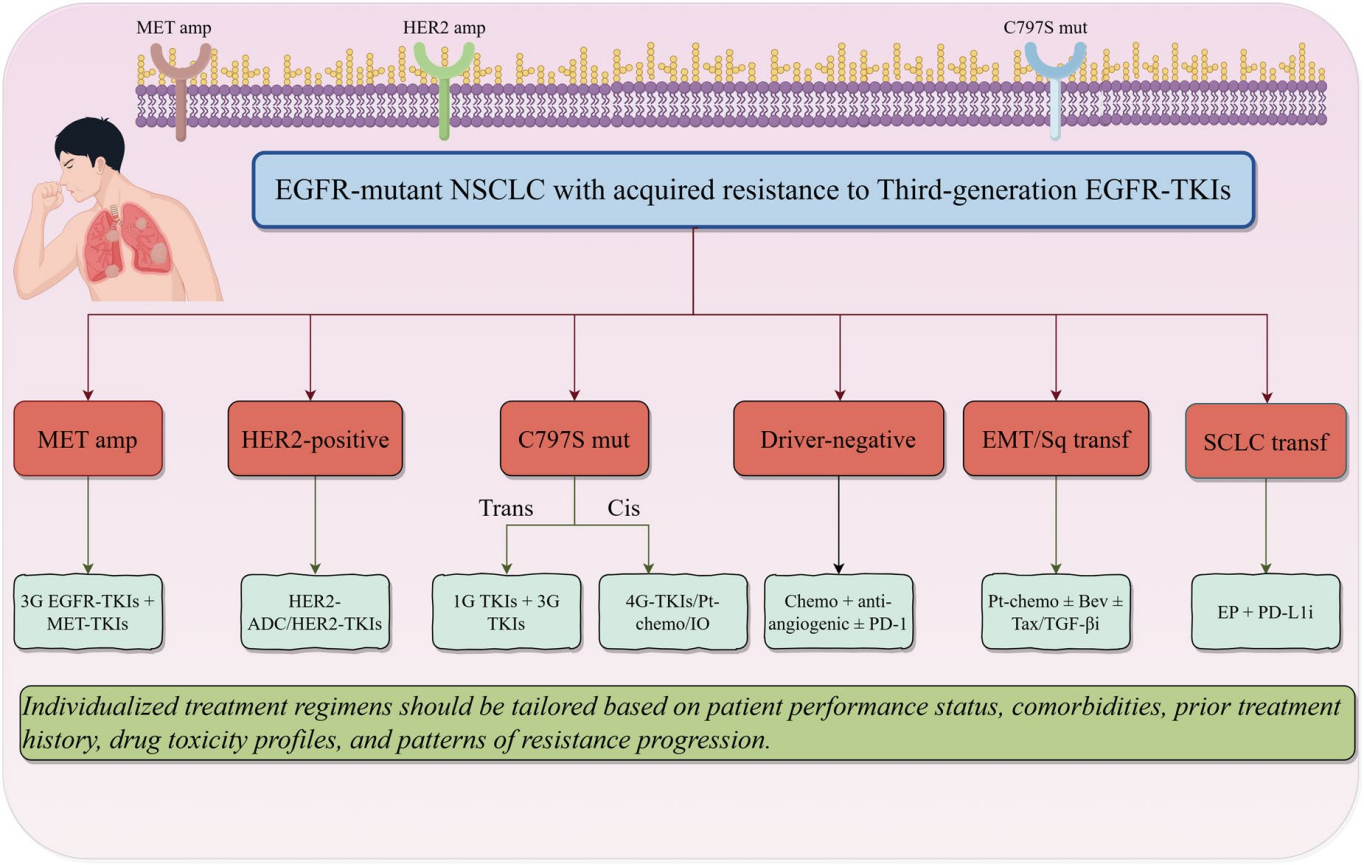
Stratified treatment pathway map for EGFR-TKI resistant NSCLC.

Implementing a stratified treatment pathway guided by comprehensive molecular characterization of acquired resistance mechanisms represents a cornerstone of precision oncology for EGFR-mutant NSCLC progressing on TKIs. This approach entails the sequential identification of the dominant resistance driver to inform evidence-based therapeutic selection: patients exhibiting T790M positivity following progression on first- or second-generation TKIs should receive third-generation EGFR TKIs such as osimertinib; conversely, those developing C797S-mediated resistance post-third-generation TKI therapy require nuanced strategies contingent upon mutation configuration—the trans conformation may warrant a combined approach using first- and third-generation TKIs despite limited clinical validation, whereas the cis conformation necessitates consideration of investigational fourth-generation TKIs within clinical trials, MET- or HER2-targeted combination regimens if concurrent amplification is detected, or conventional chemotherapy/chemoimmunotherapy. Isolated MET amplification should prompt combination therapy with an EGFR TKI and MET inhibitor (e.g. osimertinib plus savolitinib), while HER2 alterations (mutations/amplifications) may be addressed with HER2-directed agents such as T-DXd, particularly for HER2-mutant disease. SCLC transformation mandates treatment with etoposide-platinum (EP) chemotherapy combined with PD-L1 inhibitors like atezolizumab, whereas tumors manifesting EMT or squamous transformation potentially respond to bevacizumab/paclitaxel regimens with or without investigational agents targeting pathways such as TGF-β. For cases with compound mutations or undetermined resistance mechanisms, enrollment in fourth-generation TKI trials, combination chemoimmunotherapy, or cytotoxic chemotherapy represent viable options. Within each molecularly defined pathway, optimization of treatment selection requires integration of key clinical parameters including patient performance status, disease progression pattern (oligoprogressive versus systemic), prior therapy exposure, comorbidities, and comparative toxicity profiles of available interventions, with structured decision-tree models incorporating these multidimensional factors serving as valuable tools to standardize clinical decision-making in complex resistance scenarios (Figure 2) [66].

### 6.2. Optimizing clinical trial strategies

The development of efficient clinical trial methodologies is imperative to address the escalating complexity of molecular resistance mechanisms in oncology, with master protocol frameworks encompassing umbrella and basket trial designs emerging as pivotal methodological innovations. These paradigms systematically screen patient populations for predefined biomarkers and molecularly defined resistance pathways, subsequently allocating participants to parallel therapeutic sub-studies investigating matched targeted interventions based on their molecular profiles. The ORCHARD platform (NCT03944772) exemplifies this approach within the context of acquired resistance following osimertinib therapy in EGFR-mutant NSCLC: patients undergo comprehensive genomic profiling to identify the dominant resistance driver, enabling precise allocation into biomarker-guided treatment cohorts—such as those exhibiting MET amplification receiving combination therapy with savolitinib plus osimertinib, those harboring HER2 amplification administered T-DXd, or those manifesting the C797S mutation assigned to investigational fourth-generation EGFR TKIs—thereby optimizing therapeutic matching while simultaneously facilitating accelerated drug evaluation across multiple resistance subtypes. This integrated trial architecture enhances operational efficiency by consolidating biomarker screening and intervention assignment within a unified protocol, expands patient access to genomically directed therapies, and generates high-fidelity, mechanism-specific efficacy data with reduced timelines compared to conventional single-arm study designs, collectively advancing the rational development of resistance-tailored treatment strategies (Table 1).

**Table 1.** Targeted therapeutic strategies for key EGFR-TKI resistance mechanisms.

Resistance Mechanism	Representative Therapeutic Strategy	Key Agent(s)	Key Clinical Data/Evidence	Status/Notes
T790M (post 1st/2nd-gen TKI)	3rd-gen EGFR-TKI	Osimertinib	ORR: ∼70%; mPFS: 10–11 mo; (FLAURA)	Approved (global)
C797S (trans)	1st-gen + 3rd-gen EGFR-TKI	Gefitinib/Nilotinib + Osimertinib	ORR: ∼50–60% (case series/Retrospective study)	Limited evidence
C797S (cis)	4th-gen EGFR-TKI	BLU-945; BLU-701; OBX02-011; CH7233163	Early antitumor activity (Phase I)	Ongoing clinical trials
MET amplification	3rd-gen EGFR-TKI + MET-TKI	Osimertinib + Savolitinib	ORR: ∼56%; mPFS: ∼7.4 mo (SAVANNAH)	Conditionally approved (China)
HER2 mutation/amplification	HER2-ADC	Trastuzumab Deruxtecan	ORR: ∼55% (DESTINY-Lung01/02)	Approved for HER2-mutant NSCLC
SCLC transformation	EP + PD-L1	Etoposide + platinum + Atezolizumab	mOS: ∼13 mo (retrospective analysis)	Standard for de novo SCLC
SCLC (DLL3 high)	DLL3-targeted BiTE	Tarlatamab	ORR: ∼40% (Phase I)	Ongoing clinical trials
EMT/squamous transformation	Chemo ± anti-angiogenic + exploratory TKI	Paclitaxel + Bevacizumab + TGF-β inhibitor	DCR: ∼60–70%; retrospective	Anecdotal/exploratory therapy
Unknown/compound mutations	Chemo/immunochemotherapy/trials	Platinum doublet ± ICI/ 4th-gen TKI trial	Variable responses	Individualized (PS & progression)

1st-gen: first-generation; 2nd-gen: second-generation; 3rd-gen: third-generation; 4th-gen: fourth-generation; EGFR-TKI: epidermal growth factor receptor tyrosine kinase inhibitor; MET-TKI: mesenchymal–epithelial transition tyrosine kinase inhibitor; HER2: human epidermal growth factor receptor 2; ADC: antibody–drug conjugate; BiTE: bispecific T-cell engager; EP chemo: etoposide plus platinum chemotherapy; PD-L1 inhibitor: programmed death-ligand 1 inhibitor; ICI: immune checkpoint inhibitor; ORR: objective response rate; mPFS: median progression-free survival; mOS: median overall survival; mo: months; PS: performance status; SCLC: small cell lung cancer; NSCLC: non–small cell lung cancer; DLL3: delta-like ligand 3; EMT: epithelial–mesenchymal transition; TGF-β: transforming growth factor-β.

### 6.3. Toxicity management bottlenecks

Effective management of novel therapeutic agents requires vigilant toxicity mitigation strategies. For the EGFR/MET bispecific antibody amivantamab, ILD/pneumonitis occurs in 3-8% of patients (grade ≥3: 2-3%), necessitating regular symptom assessments and radiographic monitoring, with management involving prompt corticosteroid initiation and protocol-based dose modifications based on severity [31]. ADCs demonstrate characteristic class-specific toxicities: HER2/HER3-targeted ADCs commonly induce hematological adverse events (neutropenia, thrombocytopenia, anemia) requiring routine blood monitoring and supportive interventions like growth factors, while ILD represents a shared risk requiring pulmonary surveillance analogous to amivantamab. A proactive paradigm integrating scheduled monitoring, patient education on early symptom recognition, multidisciplinary management, and timely intervention according to established algorithms is essential to maintain treatment continuity and optimize safety outcomes.

### 6.4. Emerging scientific challenges

Therapeutic limitations for EGFR compound mutations, particularly the clinically challenging C797S + L798I configuration, represent a critical unmet need due to structural interference with conventional EGFR-TKI binding that substantially compromises drug efficacy. Overcoming this requires development of novel targeted agents through in-depth biological characterization of mutant-specific vulnerabilities, with key approaches including: (1) identification and targeting of critical downstream signaling nodes like hyperactivated MAPK/ERK and PI3K/AKT pathways driven by compound mutations; (2) rational dual-inhibition strategies designed to concurrently disrupt C797S- and L798I-mediated signaling cascades; and (3) engineering next-generation mutant-selective inhibitors with optimized binding affinity. Preclinical evidence indicates agents such as BDTX-1535 can inhibit >50 rare EGFR mutations including C797S and L718Q, though efficacy against L798I-containing variants requires further validation. Proactive prevention of resistance to emerging fourth-generation TKIs necessitates strategic interventions during drug development, beginning with mechanistic profiling using advanced *in vitro* and *in vivo* models to map primary and adaptive resistance pathways including MET amplification and HER3 upregulation, complemented by structural optimization through covalent warhead modification or allosteric site engagement—as exemplified by BLU-945′s activity against diverse C797S cis/trans configurations. Barrier strategies should incorporate combination therapies co-administering TKIs with escape-route inhibitors such as MET/HER2-directed agents or antibody-drug conjugates including HER3-DXd, with emerging clinical data demonstrating patritumab deruxtecan achieving 39% objective response rates in osimertinib-resistant NSCLC, alongside dynamic monitoring utilizing ctDNA-based minimal residual disease detection to enable early identification of emergent resistance clones.

## 7. Conclusion

In this review, we have mapped the complex landscape of EGFR‐TKI resistance in NSCLC—structuring its molecular underpinnings (on-target mutations, bypass activations, histologic shifts, metabolic rewiring), proposing a clinically actionable progression classification (local, oligoprogressive, systemic), and surveying state-of-the-art detection modalities from tissue and liquid biopsies to multi-omics integration and AI-driven early warning models. By aligning specific resistance drivers (e.g. T790M, C797S configurations, MET/HER2 amplifications, SCLC transformation) with matched therapeutic strategies—including combinatorial regimens, novel fourth-generation inhibitors, bispecific antibodies, and ADCs—this framework equips clinicians to personalize sequential treatment and optimize patient outcomes.

Looking ahead, key priorities include refining real-time resistance surveillance through single-cell and spatial multi-omics, accelerating development of cis-C797S inhibitors, and embedding master-protocol trials to streamline biomarker-driven therapy evaluation. With continued integration of dynamic molecular profiling, innovative drug design, and adaptive clinical platforms, we are poised to transform EGFR-mutant NSCLC from an inevitably refractory disease into a chronically manageable condition with durable control.

## Supplementary Material

highlights.docx

## Data Availability

This manuscript is a review article and does not contain any new data. Data sharing is therefore not applicable to this article.
