# Supplementary material for: Precision navigation through the labyrinth: overcoming EGFR resistance in non-Small cell lung cancer
Source: Ann Med. 2025 Oct 15;57(1):2574526. doi: 10.1080/07853890.2025.2574526 (PMC12529746; doi:10.1080/07853890.2025.2574526)
Supplement: highlights.docx [file IANN_A_2574526_SM2861.docx]

- Integrated a comprehensive panoramic atlas of EGFR-TKI resistance by categorizing molecular events into four modules—on-target secondary/tertiary mutations (T790M, C797S, L718Q), bypass pathway activations (MET/HER2 amplifications), histologic transformations, and metabolic reprogramming—to delineate the full NSCLC resistance landscape
- Established a three-tiered clinical classification system—local progression, oligoprogression, and systemic progression—and formulated operational guidelines for personalized treatment pathways based on key molecular features such as MET amplification and T790M/C797S co-occurrence
- Reviewed and integrated tissue and liquid biopsy techniques, multi-omics (genomic/epigenomic/metabolomic) fusion analyses, and AI-driven early warning models to propose a dynamic monitoring and precision intervention framework for real-time tracking and proactive management of EGFR-TKI resistance
